# Superficial Macula Capillary Complexity Changes Are Associated With Disability in Neuromyelitis Optica Spectrum Disorders

**DOI:** 10.3389/fneur.2021.724946

**Published:** 2021-09-22

**Authors:** Ruili Wei, Jianyang Xie, Huihui Wu, Fangping He, Fangxia Meng, Jiang Liu, Hui Liang, Yitian Zhao

**Affiliations:** ^1^Neurology Department, First Affiliated Hospital, Zhejiang University School of Medicine, Hangzhou, China; ^2^Cixi Institute of Biomedical Engineering, Ningbo Institute of Materials Technology and Engineering, Chinese Academy of Sciences, Ningbo, China; ^3^Department of Computer Science and Engineering, Southern University of Science and Technology, Shenzhen, China

**Keywords:** neuromyelitis optica spectrum disorders, optic neuritis, capillaris, optical coherence tomography angiography, expanded disability status scale

## Abstract

**Purpose:** We examined the macular microvascular changes of the macula in neuromyelitis optica spectrum disorder (NMOSD) patients and its association with their disability and other clinical variables.

**Methods:** Thirty-four NMOSD (13 patients without optic neuritis, NMOSD-NON, and 21 patients with a history of optic neuritis, NMOSD-ON) and 44 healthy controls (HCs) were included in the study. Optical coherence tomographic angiography (OCTA) was used to image the superficial (SCP), deep (DCP), and whole capillary plexus (WCP) in a 2.5-mm-diameter concentric circle [excluding the foveal avascular zone (FAZ)]. An algorithm (D_box_) was used to quantify the complexity of the three capillary layers by fractal analysis. We also evaluated the expanded disability scale status (EDSS).

**Results:** D_box_ values were significantly reduced in SCP (*p* < 0.001), DCP (*p* < 0.001), and WCP (*p* = 0.003) of NMOSD when compared with HCs. D_box_ values were significantly reduced in NMOSD eyes with optic neuritis when compared with healthy controls (*p* < 0.001) and eyes without optic neuritis (*p* = 0.004) in the SCP. In the DCP, eyes with optic neuritis showed significantly reduced D_box_ values when compared with eyes without optic neuritis (*p* = 0.016) and healthy controls (*p* < 0.001); eyes without optic neuritis showed significantly reduced D_box_ values (*p* = 0.007) in the DCP when compared with healthy controls. A significant negative correlation (Rho = −0.475, *p* = 0.005) was shown between the superficial macula D_box_ values and the EDSS in NMOSD patients. Additionally, a negative correlation (Rho = −0.715, *p* = 0.006) was seen in the superficial D_box_ values in [e]eyes without optic neuritis and EDSS.

**Conclusions:** Macular microvascular damage in the superficial plexus is associated with disability in NMOSD. Macular microvascular alterations arise independently of the occurrence of ON in NMOSD.

## Introduction

Neuromyelitis optica spectrum disorder (NMOSD) is an autoimmune inflammatory disorder of the central nervous system (CNS). NMOSD may target the optic nerve and spinal cord resulting in blindness and paralysis (1). Occasionally, NMOSD may target the brainstem or brain involvement (2–4), which may result in different symptoms of which severe pain (5), cognitive impairment (6), and depression (7) are common. The discovery of aquaporin-4 (AQP4) has enabled a deeper understanding of the pathological process in NMOSD and made its diagnosis much easier (8); additionally, the discovery of AQP4 has helped to distinguish NMOSD from multiple sclerosis (MS) (9). Previous reports have shown that NMOSD causes neurodegeneration and destruction of astrocytes, which leads to severe disability (10, 11).

Optic neuritis is repeatedly severe, and recovery is generally poor in NMOSD patients; as such, permanent damage increases with each inflammatory attack leading to increasing disability (12). Visual impairment, a foremost clinical manifestation in NMOSD, is responsible for severe disability and reduced quality of life (13). The retinal microvasculature is an early and prevalent target of inflammatory attack in NMOSD (14). The alterations in the retinal microvascular network of NMOSD are seen as an indicator of retinopathy onset and development (14–16). Fractal analysis has been reported to provide insight into the development of retinal vascular changes during the disease mechanism (17, 18). The emergence of optical coherence tomography angiography (OCTA) has enabled the non-invasive imaging and visualization of the retinal microvasculature in different layers. Previous reports have shown the retinal changes associated with NMOSD (14, 16, 19), and some have suggested that the retinal damage is associated with its cerebral damage (20). Recent reports (14, 15, 19) have shown that the retinal vasculature is sensitive to the inflammatory attack, and some have suggested it to be a reliable structural biomarker.

Disability has been reported to be an independent predictor of poor quality of life (QOL) in NMOSD (21); however, the macular microvascular changes associated with a disability are underexplored. Our current study aimed to explore macular microvascular changes in NMOSD and its association with the disability in NMOSD patients. These discoveries could be diagnostically beneficial and could have inferences for understanding the pathogenesis of NMOSD.

## Methods

This study enrolled 36 NMOSD patients and 44 healthy controls. NMOSD patients were enrolled from the Neurology Department of the First Affiliated Hospital, College of Medicine, Zhejiang University, China. The diagnosis of NMOSD patients was based on the revised diagnostic criteria of Wingerchuk et al. (1). All NMOSD patients enrolled in our study were positive for the anti-AQP4 antibody and did not have optic neuritis in the last 6 months. Clinical data, such as disease duration, frequency of optic neuritis, expanded disability status scale (EDSS) and visual acuity, were obtained. Ethical approval for the study was obtained from the institutional Ethics Committee of the First Affiliated Hospital, College of Medicine, Zhejiang University, Hangzhou, China. Written informed consent was obtained from each participant, and the protocols of the study were under the Declaration of Helsinki.

Sex- and age-matched healthy controls were recruited from participants who had their annual health checkup at our hospital. Subjects were excluded if they had a systemic or neurological disease that could affect the brain and retina.

### Measurement of Visual Acuity

Snellen charts at a 2.5-m distance were used as the measurement of visual acuity of each participant, and finger counting [later transformed as visual acuity (22)] was done for patients with poor vision. Visual acuity was transformed to the logarithm of the minimum angle of resolution (LogMAR) for statistical analyses.

### Macular Microvascular Imaging

Imaging of the macular microvascular was done with the OCTA tool (Cirrus 5000, V.10.0; Zeiss Meditec, CA, USA). The OCTA used a wavelength of 840 nm and an A-scan rate of 68,000 scans per second. Participants were asked to focus on the center of the target in the machine to allow imaging. Imaging of the macula microvasculature was done at the macula with a 3 × 3-mm scan pattern.

For the inclusion criteria in our present study, high-quality images with a signal strength index ≥6 were accepted according to the OSCAR-IB criteria (23).

### Quantitative Analysis of the Macular Microvasculature Based on Fractal Dimension

**Figure 1:**
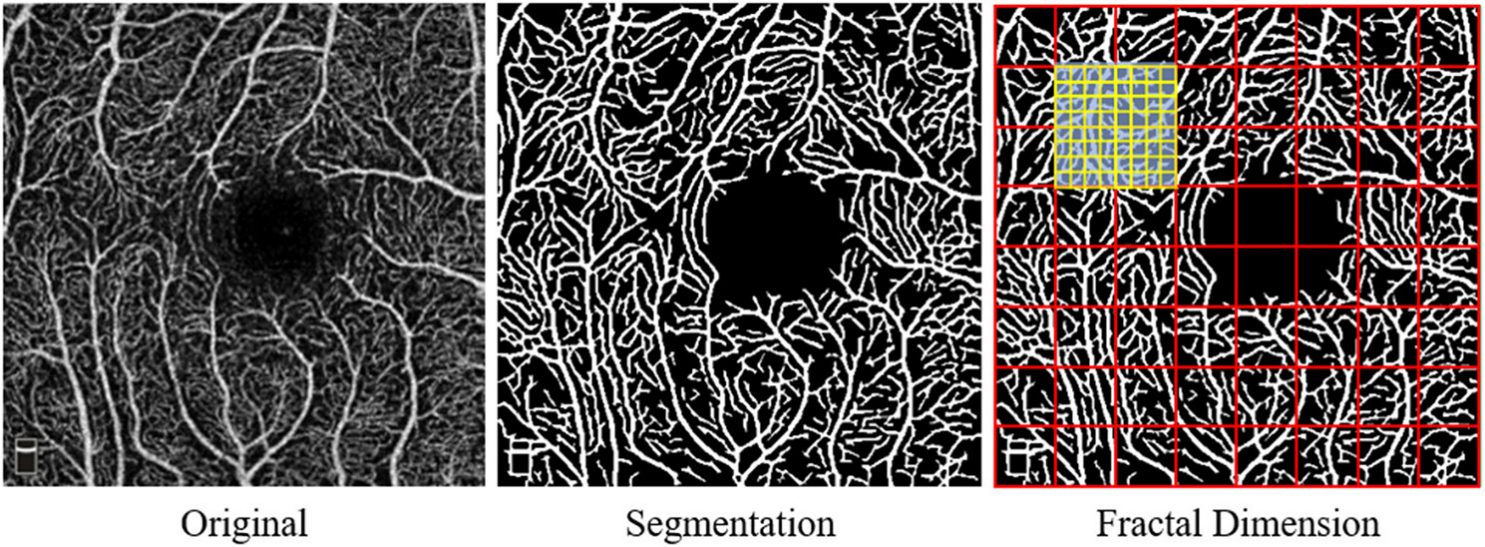
Display of the macular capillary plexus and its quantitative analysis using fractal dimension (FD).

Quantitative analysis of the macular microvascular fractal dimension (FD) at the superficial capillary plexus (SCP), deep capillary plexus (DCP), and whole capillary plexus (WCP) was done using the *en face* OCTA images (PNG format) as shown in Figure 1. The superficial retinal capillary plexus (SCP) extended from 3 μm below the internal limiting membrane (ILM) to 15 μm below the inner plexiform layer (IPL); the deep retinal capillary plexus (DCP) extended from 15 to 70 μm below the IPL. The WCP was 3 μm below the internal limiting membrane to the base of the outer plexiform layer (OPL). A parafoveal capillary network was acquired through 3 × 3-mm^2^ scans within the annular zone of 0.6–2.5-mm diameter around the foveal center. FD measurement was fully automated and implemented on a vasculature segmentation map. To obtain the binary microvasculature network, the U-net framework, which contains an encoder and a decoder component was applied on each two-dimensional OCT-A grayscale image. The used U-net model was pretrained in the ImageNet and then adjusted through our OCTA dataset where the vessel standard was annotated by image experts in SCP, DCP, and WCP layers, respectively (http://www.image-net.org/download-images). The boundary foveal avascular zone (FAZ) was detected in all three capillary plexus layers by the U-net model and later generated binary images of the microvasculature. Based on these binary images, a skeletonized image was created.

After that, the fractal analysis was done on skeletonized images of the superficial, deep, and whole capillary plexus. The box-counting method was used on each macular microvascular segmentation map to calculate the fractal dimension (FD). Box counting (D_box_ values) from Fraclab (https://www.project.inria.fr/fraclab/) was applied to quantify the complexity in all three capillary plexuses (i.e., SCP, DCP, and WCP) in the 2.5-mm diameter of the fovea. The methods above were implemented using MATLAB 2016b (Mathworks, Inc., Natick, MA, USA) (24).

### Statistical Analyses

Statistical analyses were done using SPSS software (SPSS, version 24). Student's *t*-test or Chi-square test was performed to compare groups depending on the normality and properties of the variables. A generalized estimation equation was used for all analyses between and among groups while adjusting for age, gender, signal quality (SQ) of the angiogram, and inter-eye dependencies from the same participant. Pearson's correlation was used to assess correlations between OCTA measures and clinical parameters and after GEE was used while adjusting for signal quality of OCTA images. A value of *p* < 0.05 represents statistical significance.

## Results

We initially enrolled 46 NMOSD patients, but 12 were excluded due to poor image quality (SQ < 6). Thirty-four NMOSD patients (mean age: 52.48 ± 12.99 years) and 44 healthy controls (mean age: 53.11 ± 11.83 years) who were sex- and age-matched were included for data analyses. Out of the 34 NMOSD patients, 13 (38.2%, Table 1) had no history of optic neuritis, 10 (26.3%, Table 1) had experienced optic neuritis once, while 11 (35.5%, Table 1) had experienced optic neuritis twice or more. The mean disease duration of the NMOSD patients was 7.2 ± 5.3 months, while the frequency of optic neuritis was 2.48 ± 1.91 times. Our NMOSD patients were divided into two groups according to the occurrence of optic neuritis: NMOSD without optic neuritis, NMOSD-NON, and NMOSD without optic neuritis, NMOSD-ON. The clinical variable of our participants is shown in Table 1.

**Table 1:** Demographics and clinical variables.

	**NMOSD group**	**Control group**
Number	34	44
Gender (female:male)	32:2	41:3
Age, years	52.48 ± 12.99	53.11 ± 11.83
Frequency of ON	2.48 ± 1.91	–
**Optic neuritis**		
0	13	–
1	10	–
≥2	11	–
Mean disease duration, months	7.2 ± 5.3	–
EDSS, mean	2.57 ± 1.16	–
Visual acuity, LogMAR	0.510 ± 0.70	−0.057 ± 0.06

*NMOSD, neuromyelitis optica spectrum disorder; ON, optic neuritis; EDSS, expanded disability scale status; LogMAR, logarithm of the minimum angle of resolution*.

VA did not differ (*p* = 0.063) in NMOSD patients without optic neuritis when compared with healthy controls, but a significant difference was seen in NMOSD patients with optic neuritis when compared with healthy controls (*p* < 0.001).

### Fractal Analysis Using D_box_ Between Euromyelitis Optica Spectrum Disorder and Healthy Controls

D_box_ values were significantly reduced in the SCP (*p* < 0.001, Table 2), DCP (*p* < 0.001, Table 2), and WCP (*p* = 0.003, Table 2) of NMOSD patients when compared with healthy controls.

**Table 2:** Comparison of macula microvascular fractal dimension (FD) between NMOSD and healthy controls (HCs).

	**NMOSD**	**HC**	***p*-Value**
Superficial capillary plexus (SCP)	1.39 ± 0.43	1.43 ± 0.23	<0.001
Deep capillary plexus (DCP)	1.42 ± 0.56	1.48 ± 0.38	<0.001
Whole capillary plexus (WCP)	1.43 ± 0.33	1.45 ± 0.22	0.003

*p-Values were adjusted for age, gender, the signal value of angiogram, and intereye dependencies*.

**Figure 2:**
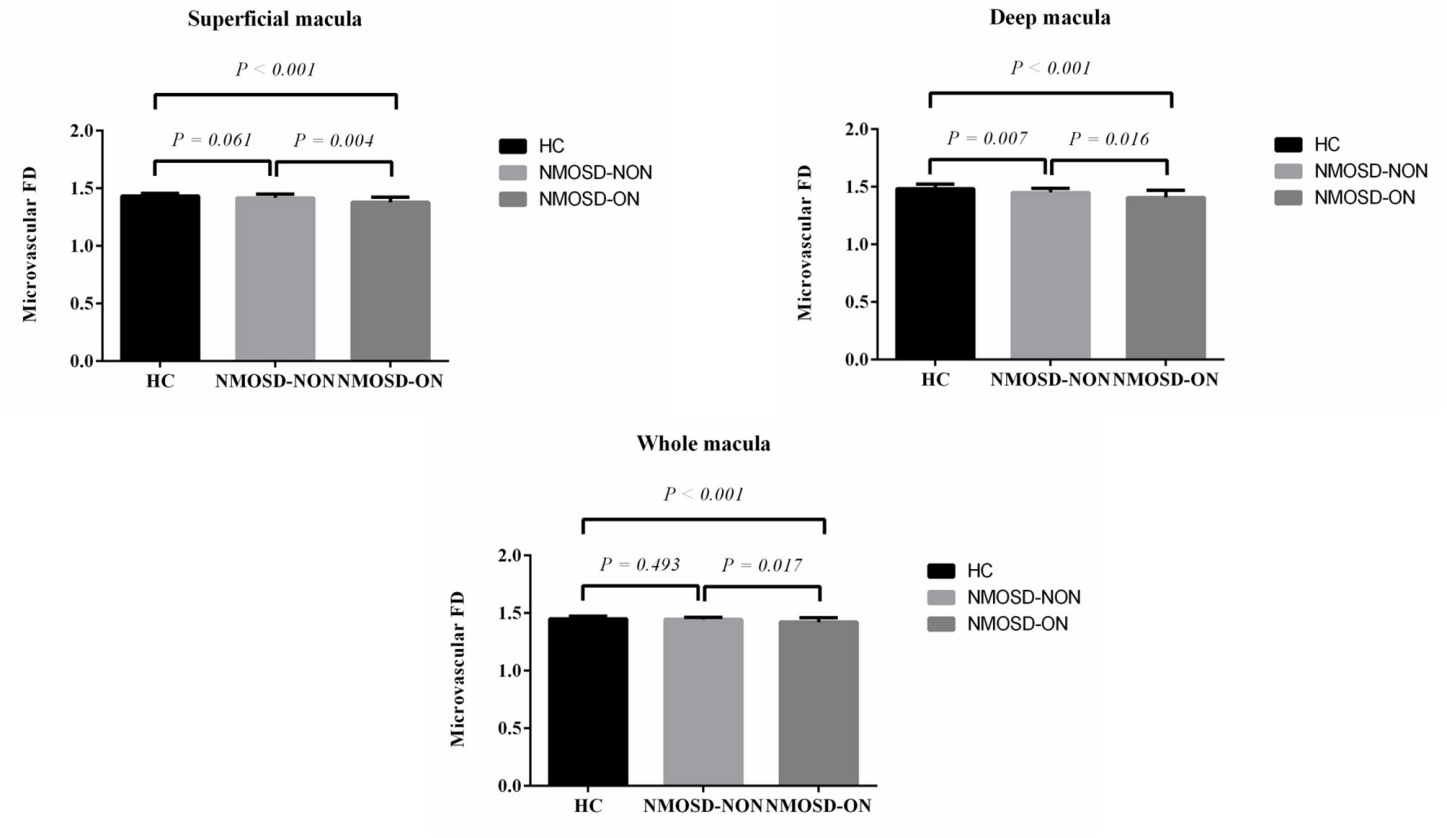
Comparison of macular FD among neuromyelitis optica spectrum disorder (NMOSD) eyes with and without optic neuritis and healthy controls (HC).

D_box_ values were significantly reduced in the SCP of NMOSD eyes with optic neuritis when compared with healthy controls (*p* < 0.001, Figure 2) and NMOSD eyes without optic neuritis (*p* = 0.004, Figure 2). In the DCP, eyes with optic neuritis showed significantly reduced D_box_ values when compared with eyes without optic neuritis (*p* = 0.016, Figure 2) and healthy controls (*p* < 0.001, Figure 2); NMOSD eyes without optic neuritis showed significantly reduced D_box_ values (*p* = 0.007, Figure 2) in the DCP when compared with healthy controls. In the WCP, eyes with optic neuritis showed significantly reduced D_box_ values when compared with healthy controls (*p* < 0.001, Figure 2) and eyes without optic neuritis (*p* = 0.017, Figure 2).

### Association Between Macula Microvessels and Clinical Variables

**Figure 3:**
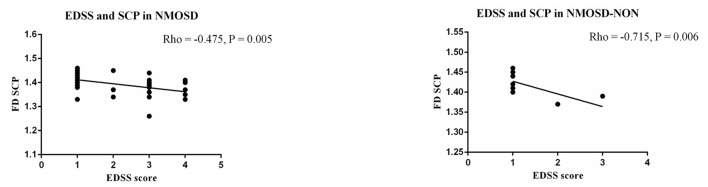
Correlation between expanded disability scale status (EDSS) and macula FD in NMOSD patients.

A significant negative correlation (Rho = −0.475, *p* = 0.005; Figure 3) was shown between the SCP D_box_ values and the EDSS in all NMOSD patients. Additionally, a negative correlation (Rho = −0.715, *p* = 0.006; Figure 3) was seen in the SCP D_box_ values in eyes without optic neuritis and EDSS. However, no significant correlation was seen between EDSS and the SCP (Rho = −0.221, *p* = 0.336), DCP (Rho = −0.085, *p* = 0.714), and WCP (Rho = −0.001, *p* = 995) in NMOSD eyes with optic neuritis.

A significant positive correlation (Rho = 0.378, *p* = 0.011) was seen between the visual acuity and D_box_ values in the WCP of healthy controls. A significant negative correlation (Rho = −0.445, *p* = 0.008) was seen between the visual acuity and D_box_ values in the SCP of NMOSD patients.

There was no significant correlation between EDSS and visual acuity in the entire NMOSD group (Rho = 0.477, *p* = 0.053). No significant correlation (*p* > 0.05) was seen between the EDSS and visual acuity in the NMOSD subgroups.

## Discussion

To our knowledge, this is the first study to use fractal analysis of OCTA images to evaluate the macular microvasculature complexity in NMOSD and explore the association between the macular microvascular changes and their clinical implication. Our study showed that D_box_ values were significantly reduced in the SCP, DCP, and WCP in the eyes with optic neuritis when compared with eyes without optic neuritis. Additionally, we found that the reduced D_box_ value in the SCP was significantly associated with the EDSS in NMOSD patients. Our current study suggests that fractal analysis of OCTA images can help in identifying the subclinical vasculopathy in NMOSD before the occurrence of the clinically apparent optic neuritis.

Macular microvascular changes seen in our NMOSD patients when compared with healthy controls are congruent with previous OCTA reports (14–16). A noteworthy finding was that eyes without optic neuritis showed a significantly reduced D_box_ value in the DCP when compared with healthy controls. This finding suggests that early macular microvascular alterations associated with NMOSD may first occur in the DCP because this capillary plexus contains capillaries that are smaller and prone to slight changes (25). The Dbox values in eyes with ON were significantly reduced compared with eyes without ON in the superficial, deep, and whole macular microvascular plexus. This may suggest that the amount of dropoff in macular microvascular complexity is linked with and foretells the development of retinopathy in NMOSD after the inflammatory attack(s).

A novel finding in our current report is the association between EDSS and macular microvascular damage in the SCP. EDSS is a method of quantifying disability in NMOSD; furthermore, it has been reported that disability is an independent predictor of poor QOL in NMOSD (21). Previous reports (26–28) on multiple sclerosis (MS) found an inverse correlation between ganglion cell layer–inner plexiform layer (GCIP) thickness and EDSS; these reports suggested that neuronal damage in this portion of the retina reflect the disability of patients in MS. Likewise, the significant inverse correlation between EDSS and macular microvascular damage in NMOSD patients could suggest that the microvascular damage in the SCP, which is found within the retinal nerve fiber layer (RNFL), ganglion cell layer–inner plexiform layer, may affect the disability in NMOSD patients. EDSS scores are rough estimates of clinical status and are weighted toward locomotor incapacity, which is likely dependent on the damage of the spinal cord as previously reported (29). Our report suggests that macular microvascular damage in the SCP may be associated with damage in the spinal cord, and further studies are needed to validate our hypothesis. Besides, the significant inverse correlation between the EDSS and SCP microvascular damage in NMOSD without optic neuritis could also suggest the same hypothesis stated above.

Our study showed a significant correlation under normal conditions between the whole microvasculature and visual acuity indicating that the integrity of the microvasculature contributes to the visual function in a healthy individual. Remarkably, our data showed that a reduction in the Dbox value in the SCP was associated with poor visual acuity in NMOSD patients. Sotirchos et al. (30) suggested that the anterior visual pathway is significantly involved in the subclinical phase of NMOSD; since SCP consists of the sublayers of the retina (retinal ganglion cells, RGCs), we suggest that our current data are congruent with the aforementioned report.

Our study had some limitations. First, the cross-sectional study does not yet prove that microvascular changes seen on the OCTA are necessarily associated with disability and prognosis; longer-term follow-up is needed to demonstrate the correlation between disability progression and retinal microvasculature damage. Also, the cross-sectional study limits our understanding on whether these microvascular changes are comprehensive ischemia or due to changes in the retinal structure. Thus, longitudinal studies are required to warrant our hypotheses on the microvascular changes.

In conclusion, our results indicated that fractal dimensions decrease in NMOSD patients with or without optic neuritis when compared with healthy controls. A significant decrease in the deep capillary plexus in the eyes without optic neuritis shows that macular microvascular damage in the deep plexus may occur before optic neuritis. A novel finding in our report was the possible association between macula microvascular damage in the superficial plexus and disability in NMOSD. We suggest that macular microvascular alterations arise independently of the occurrence of ON in NMOSD.

## Data Availability Statement

The raw data supporting the conclusions of this article will be made available by the authors, without undue reservation.

## Ethics Statement

The studies involving human participants were reviewed and approved by First Affiliated Hospital, Zhejiang University School of Medicine. The patients/participants provided their written informed consent to participate in this study.

## Author Contributions

All authors listed have made a substantial, direct and intellectual contribution to the work, and approved it for publication.

## Funding

This work was financially supported by Administration of traditional Chinese medicine project of Zhejiang Province (2018ZA067).

## Conflict of Interest

The authors declare that the research was conducted in the absence of any commercial or financial relationships that could be construed as a potential conflict of interest.

## Publisher's Note

All claims expressed in this article are solely those of the authors and do not necessarily represent those of their affiliated organizations, or those of the publisher, the editors and the reviewers. Any product that may be evaluated in this article, or claim that may be made by its manufacturer, is not guaranteed or endorsed by the publisher.
